# Pectoral muscle identification in mammograms

**DOI:** 10.1120/jacmp.v12i3.3285

**Published:** 2011-03-03

**Authors:** K. Santle Camilus, V. K. Govindan, P.S. Sathidevi

**Affiliations:** ^1^ Department of Computer Science and Engineering National Institute of Technology Calicut Calicut India; ^2^ Department of Electronics and Communication Engineering National Institute of Technology Calicut Calicut India

**Keywords:** biomedical image analysis, pectoral muscle identification, mammogram analysis, computer‐aided detection, segmentation

## Abstract

In most of the approaches of computer‐aided detection of breast cancer, one of the preprocessing steps applied to the mammogram is the removal/suppression of pectoral muscle, as its presence within the mammogram may adversely affect the outcome of cancer detection processes. Through this study, we propose an efficient automatic method using the watershed transformation for identifying the pectoral muscle in mediolateral oblique view mammograms. The watershed transformation of the mammogram shows interesting properties that include the appearance of a unique watershed line corresponding to the pectoral muscle edge. In addition to this, it is observed that the pectoral muscle region is oversegmented due to the existence of several catchment basins within the pectoral muscle. Hence, a suitable merging algorithm is proposed to combine the appropriate catchment basins to obtain the correct pectoral muscle region. A total of 84 mammograms from the mammographic image analysis database were used to validate this approach. The mean false positive and mean false negative rates, obtained by comparing the results of the proposed approach with manually‐identified (ground truth) pectoral muscle boundaries, respectively, were 0.85% and 4.88%. A comparison of the results of the proposed method with related state‐of‐the‐art methods shows that the performance of the proposed approach is better than the existing methods in terms of the mean false negative rate. Using Hausdorff distance metric, the comparison of the results of the proposed method with ground truth shows low Hausdorff distances, the mean and standard deviation being 3.85±1.07 mm.

PACS numbers: 87.57.R, 87.57.nm, 87.59.ej, 87.85.Ng, 87.85.Pq

## I. Introduction

Due to the drastic growth in mammography, a huge number of higher quality and diverse images are available for analysis. At this juncture, usage of computer vision techniques, which includes artificial systems to analyze these medical images, is indispensable. Artificial systems should be designed to analyze medical images in a semi‐automatic or even in a fully automatic manner. However, the usage of artificial systems for mammogram analysis is not new to this field. Though computer‐aided detection (CAD) for breast cancer is available in the market, studies^(^ ^1^ ^,^ ^2^ ^)^ demonstrate that further developments are required in this field to produce more effective CAD.

A mammogram is a two‐dimensional image of a three‐dimensional breast. The intensity of each pixel of the mammogram results from the superposition of several types of tissues through which X‐rays pass. This superposition causes several problems in identifying different breast tissues, including the pectoral muscle.^(3)^ In the mammogram, the pectoral muscle appears as a triangular region in one side of the image. The breasts cover the chest muscle, namely, the pectoralis major which is attached to the collarbone, breastbone and the cartilage of most of the ribs. The pectoralis minor is a triangular‐shaped chest muscle that lies under the pectoralis major and is attached to the third, fourth, and fifth ribs. Either one of these two chest muscles is commonly called the pectoral muscle. In most of the breast cancer detection methods, removal or suppression of the pectoral muscle is a preprocessing step, as its presence within a mammogram may affect the results.^(4)^ Also, pectoral muscle identification can be used in image registration for abnormality analysis like bilateral symmetry.^(5)^ Hence, it is essential to pay attention to pectoral muscle identification so as to produce effective results in mammogram‐based lesion detections using CAD.

In the literature, several methods have been proposed to identify the pectoral muscle in mammograms. Suckling et al.^(6)^ used a multiple‐linked self‐organizing neural network to segment the pectoral muscle. However, this method requires a set of good training mammograms to generate satisfactory results. Masek et al.^(7)^ employed both a threshold‐based algorithm and a straight line fitting technique to represent the pectoral muscle. The straight line representation by this method is not an efficient way to identify the pectoral muscle due to the existence of curved pectoral muscles. A method based on the estimation and refinement of the pectoral muscle edge was suggested by Kwok et al.^(^ ^8^ ^,^ ^9^ ^)^ This method is weak in detecting textural and vertical pectoral edges. The Hough transform was first exploited by Karssemeijer^(10)^ for identifying the pectoral muscle. Following this work, many authors developed several methods in which the Hough transform was used in part.^(^ ^11^ ^,^ ^12^ ^,^ ^13^ ^)^ As the classical Hough transform is a straight line representation technique, a suitable postprocessing is essential for the precise representation of the pectoral muscle; failure to do so may lead to inaccurate results. Ferrari et al.^(4)^ utilized a set of Gabor wavelet filters that was designed to highlight the pectoral muscle edge. To produce good results using this method, one must set the filter parameters appropriately. Raba et al.^(14)^ combined both an adaptive histogram approach and a selective region growing algorithm for the pectoral muscle segmentation. The weakness of this method is segmentation leakage, in which dense breast tissues are included falsely in the pectoral muscle region. Bajger et al.^(15)^ and Fei Ma et al.^(^ ^16^ ^,^ ^17^ ^)^ suggested two graph‐based approaches. The first approach was based on the minimum spanning tree (MST) method, while the second approach was based on the adaptive pyramid (AP) method. The MST approach failed to identify the smaller volume pectoral muscles.^(17)^ Camilus et al.^(18)^ presented an approach using a graph cut based merging method and a Bezier curve algorithm. The result of this approach is influenced by the order in which regions are merged in the graph cut based merging method.

We propose an efficient approach using the watershed transformation^(19)^ for pectoral muscle identification for following reasons. Apart from one or more of the above deficiencies in existing methods, the survey of pectoral muscle identification also reveals that most of these methods (for example, Hough transform) extract pectoral muscles as straight lines. However, this assumption is always not correct, as curved pectoral muscles do exist. The watershed transformation is an intuitive method which enables the detection of pectoral muscle boundary as a curve, which is a desirable property for identifying the true boundary points of pectoral muscle. Furthermore, most of the methods in the literature (e.g., region growing and MST algorithm) give rise to incorrect results when dense tissues appear near the pectoral muscle, as they fail to discover boundaries between them. The primary reason for the failure is the occurrence of subtle low‐contrast boundaries between the tissue and the muscle. On the other hand, watershed transformation has proved to be successful in locating the boundaries of adjacent regions even when images have low contrast and weak boundaries. Thus, the watershed transformation could be a more suitable choice for pectoral muscle identification, and it overcomes the limitations of existing methods. The proposed method is based on the knowledge of shape and location of the pectoral muscle and other image information. When a mammogram is processed with the watershed transformation, the results show a strong indication of the presence of the pectoral muscle boundary with a set of properties. However, the pectoral muscle region is oversegmented (divided into many smaller regions). Hence, a novel merging algorithm, which is particularly more suitable for the pectoral muscle segmentation, is proposed to overcome this problem. The proposed merging algorithm is based on the work of Camilus et al.^(20)^ The proposed approach produced better results in terms of accuracy. We also demonstrate, in Section III below, the improved performance of the proposed approach by comparing it's accuracy with many related state‐of‐the‐art methods.

## II. MATERIALS AND METHODS

### A. Dataset

We used a dataset which consists of 84 mammograms and their ground truth, which was provided by Ferrari et al.,^(4)^ to test the performance of the proposed approach. These mammograms were originally taken from the mammographic image analysis database,^(21)^ which is a public database of mammograms available online. All the mammograms were 8‐bit gray level images, digitized at 200 micron pixel edge, and of 1024×1024 pixel size. The manually‐extracted pectoral muscle boundaries of the mammograms, done by an expert radiologist, were used as the ground truth. In fact, the same dataset and ground truth were previously used by others to validate their works. Hence, the results obtained for this dataset by using the proposed approach can be compared directly with the previous works. To minimize the time complexity, the original size of the mammogram was reduced to 256×256 pixels and processed by our approach. Once the pectoral muscle was identified, the original size was retained by up‐sampling of the results. The results were then analyzed.

### B. Overview of the proposed approach

There are two standard views for screening mammography: 1) craniocaudal (CC) view, and 2) mediolateral oblique (MLO) view. The CC is a top‐to‐bottom view of a breast, while the MLO is a side view angled approximately between 30° to 70°. When properly taken, an MLO mammogram shows all breast tissues in one image.^(22)^ The appearance of the pectoral muscle as a triangular region in one side of the mammogram is one of the most important features of the MLO view. Hence, it is required to identify the pectoral muscles in MLO mammograms, which is the main subject of this study.

This approach was designed to identify the pectoral muscle in left MLO mammograms. However, a right MLO mammogram was processed by flipping to make it look like a left MLO mammogram. Once the pectoral muscle was identified, its original position was resumed by flipping it again. This avoided the situation of designing two different methods for treating the left and the right MLO mammograms separately.

**Figure 1:**
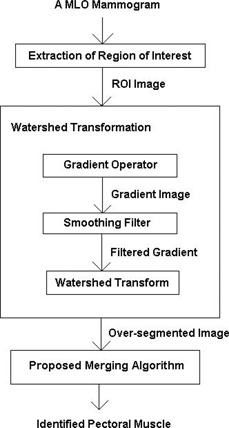
Overview of the proposed method.

In this approach, a single MLO mammogram was accepted as the input and from this, a region of interest (ROI), which holds the whole pectoral muscle, was extracted. It is a common practice to apply watershed transform to the gradient of an image instead of directly applying it to the image itself, as catchment basin boundaries can effectively be located at higher gradient points.^(23)^ Hence, a ROI gradient was obtained and filtered using a smoothing filter. The filtering step is essential to reduce the formation of several irrelevant catchment basins during the watershed transform. However, the resultant image of the watershed transform of the filtered ROI gradient was still oversegmented. Hence, the proposed merging algorithm was used to fuse the oversegmented pectoral muscle region to acquire the pectoral muscle boundary. An overview of the proposed approach is depicted in Fig. 1.

### C. Extraction of ROI

**Figure 2:**
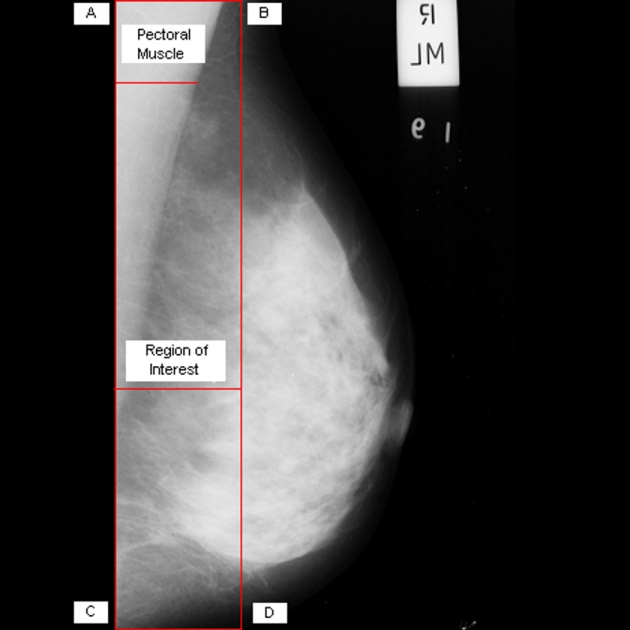
Extraction of ROI.

The left side unexposed X‐ray portion of the mammogram was completely eliminated such that the top left most pixel is a pectoral muscle pixel. Also, the skin–air boundary was approximately determined in few upper rows of the mammogram and was utilized to select the region ABDCA, in Fig. 2, as ROI which includes the complete pectoral muscle.

### D. Watershed transformation

**Figure 3:**
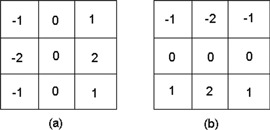
The Sobel operator: (a) $G_{x}$, (b) $G_{y}$.

The Sobel operator^(24)^ employs a pair of 3×3 convolution masks, as given in Fig. 3, to obtain the ROI gradient. The masks, $G_{x}$ (Fig. 3(a) and $G_{y}$ (Fig. 3(b), on convolving individually on the ROI, compute the horizontal and the vertical gradient approximations, respectively. At each pixel in the image, the combination of these two gradient approximations renders the gradient magnitude, which is given by:

(1) $$G=\sqrt{G_{x}^{2}+G_{y}^{2}}$$

The gradient's direction is given by:

(2) $${\text{tan}}^{-1}\left(\frac{G_{y}}{G_{x}}\right)$$

To smoothen the ROI gradient, a mean filter of 3×3 was used. The mean filter smoothes the local variations in ROI gradient and also reduces noise.^(25)^ The filtered ROI gradient was given as the input to the watershed transform. Hereafter, we call the filtered ROI gradient image the “ROI gradient”.

The watershed transform was first proposed by Diagabel and Lantuejoul.^(19)^ It is a region‐based segmentation approach from the field of mathematical morphology, and a well‐organized survey of its different definitions and algorithms can be found in the work of Roerdink and Meijster.^(26)^ The concept of watershed transform can be realized by visualizing the ROI gradient as a topographic surface, such that the gray value of each pixel defines its altitude. A hole is pierced in each regional minimum which allows water to gradually rise in catchment basins; in fact, each basin is evolved from a regional minimum. When any two catchment basins are about to merge, a dam is built between them to prevent them from merging. When water reaches the highest peak of the landscape, the flooding process is stopped. Finally, several catchment basins divided by dams (otherwise called watersheds) are evolved. In terms of image segmentation, these catchment basins represent different regions, and watersheds are the boundaries between these regions.

The ROI gradient can be viewed as a triple G=(V,E,f), where (*V, E*) is an undirected graph such that the pixels, defined in a two‐dimensional space, constitute the vertices *V*, and the edges *E* specify the connectivity between pixels. In this work, as the eight‐neighborhood connectivity of pixels was used, a vertex can have horizontal, vertical, and diagonal neighbors. In the triple, *f* is a function which assigns an integer value to every pixel, *p*∊*V*. Graph theory operations can now be applied to this graph constructed from the ROI gradient.

#### D.1 Geodesic distance

Let *a*, *b* be two points in two‐dimensional integer space, $A∊Z^{2}$. The geodesic distance between these two points within *A* is the minimum length path among all possible paths between *a* and *b* within *A*. If *B* is a subset of *A*, then the geodesic distance $d_{A}$ between *a* and *B* is

(3) $$d_{A}(a,B)=\underset{b\in B}{\text{min}}(d_{A}(a,b))$$

It is the minimum length path among all paths within *A* from the point *a* to every point of *B*.

#### D.2 Geodesic influence zone

Let *B* be contained in *A* and consist of “k” number of connected components, say $B_{i},i=1,2\ldots k$. The geodesic influence zone of $B_{i}$ within *A* is defined as:

(4) $$iz_{A}(B_{i})=\{p\in A|\forall_{j}\in [1..k]\backslash \{i\}:d_{A}(p,B_{i})<d_{A}(p,B_{j})\}$$

where “*\*” is the set difference operator. For a specific connected component, say $B_{i}$, the geodesic influence zone is a set of points of *A* which are closer to $B_{i}$ than any other connected components of *B*. The union of geodesic influence zones of all connected components of *B* is represented by a set ${\text{IZ}}_{A}$ *(B)* as:

(5) $$IZ_{A}(B)=\overset{k}{\underset{i=1}{⋃}}iz_{A}(B_{i})$$

#### D.3 Threshold set

Let *h* be a gray level between $h_{\text{min}}$ and $h_{\text{max}}$ such that

(6) $$h_{\text{min}}=\underset{p\in V}{\text{min}}(f(p))$$

(7) $$h_{\text{max}}=\underset{p\in V}{\text{max}}(f(p))$$

The threshold set of *f* at level *h*, which is defined as the set of vertices whose gray values are less than or equal to *h*, can be represented as:

(8) $$T_{h}=\{p\in V|f(p)\leq h\}$$

#### D.4 Watershed transform

Let $X_{h}$ be the union of the collection of catchment basins at level *h*, and ${\text{MIN}}_{h}$ denote the union of all regional minima at level *h*. The immersion based watershed transform, as defined by Vincent and Soille,^(27)^ is a recursive step, as follows:

(9) $$\begin{array}{l} X_{h_{\text{min}}}=\{p\in V|f(p)=h_{\text{min}}\}=T_{h_{\text{min}}}\\ X_{h+1}=MIN_{h+1}⋃IZ_{T_{h+1}}(X_{h}),\;\;\;\;\;\;h\in [h_{\text{min}},h_{\text{max}}) \end{array}$$

In the first iteration, catchment basins are formed out of the vertices having the lowest gray value. In the successive iterations (say at level h+1), either one or both of the following events occur:

(i) creation of a new set of regional minima, and/or
(ii) expansion of catchment basins of level *h*.

#### D.5 Watersheds

The watersheds are the vertices that divide the whole ROI gradient into several catchment basins. These vertices are given by:

(10) $$W_{shed}(f)=V\backslash X_{h_{\text{max}}}$$

In another words, the watersheds are the collection of vertices of the graph that excludes the vertices included in $X_{h}$ computed at $h_{\text{max}}$. In a practical sense, the watershed transformation is a process that assigns a unique label to each catchment basin and a special label to watersheds.^(26)^

#### E. The proposed merging algorithm

**Figure 4:**
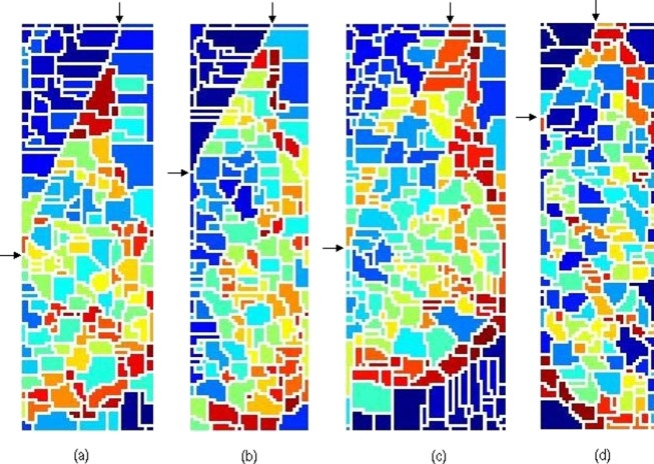
The watershed transformation of ROI gradients: (a) mdb043 (b) mdb099 (c) mdb119 (d) mdb129. For clarity, the watershed lines of interest are shown using arrows. Different colored regions represent various catchment basins. The white lines are watersheds.

When the gradient of the ROI of a mammogram was treated with the watershed transform, the results, given in Fig. 4, showed a strong indication of the presence of the pectoral muscle boundary with a set of following properties:

(i) There is a unique continuous watershed line (hereafter it is called the watershed line of interest) which starts from the top and ends at the left of the image. The width, extending from the left‐most pixel of each row (starting from the top row) to its current position, is gradually decreasing and becomes zero when it reaches the left‐most position at the end.
(ii) The watershed line of interest encloses a triangular region covering the left top region of the image. It has a curved shape.
(iii) The pectoral muscle is oversegmented; this is caused by several irrelevant regional minima within the pectoral muscle. The irrelevant regional minima may appear due to noise, local variations, etc.

Properties (i) and (ii) seem to match the pectoral muscle attributes as reported in the literature. To summarize, a few attributes of the pectoral muscle in the ROI are:

- the pectoral muscle occupies the left top corner of the image^(^ ^8^ ^,^ ^17^ ^,^ ^18^ ^)^
- the pectoral muscle forms a roughly triangular shaped region^(^ ^8^ ^,^ ^17^ ^,^ ^18^ ^)^
- from top to bottom, there is a gradual decrease in the pectoral muscle width^(^ ^17^ ^,^ ^18^ ^)^
- the pectoral muscle edge may be approximated by a curve^(^ ^8^ ^,^ ^9^ ^)^

From these observations, one can conclude that the watershed line of interest represents the pectoral muscle edge. In addition, the property (iii) establishes that by employing a suitable merging mechanism, the irrelevant regions (catchment basins) could be united to obtain the pectoral muscle region. Hence, a novel merging algorithm, which is more suitable for the pectoral muscle edge extraction, is proposed. The proposed merging algorithm is presented as pseudocode in Algorithm 1 which unifies the oversegmented catchment basins of pectoral muscle.

#### E.1 Algorithm 1: Algorithm for identifying the pectoral muscle from an oversegmented image

1. Procedure: merging‐algorithm
2. INPUT: oversegmented image
3. OUTPUT: segmented image: seed → pectoral muscle, and complement of seed → background
4. seed ← catchment basin which has a point at location (1,1) /* initial value of seed*/
5. merge ← 1
6. while merge, do:
7. for i=1 to *n* do
8. *r*(*i*) ← *InterRe gionedgeMean(seed*, $N_{i}(\text{seed}))/*N_{i}$ is the ith neighborhood basin*/
9. *d*(*i*) ← *DynamicThreshold(seed*, $N_{i}(\text{seed}))$
10. end for
11. merge ← 0
12. for i=1 to *n* do
13. if r(i)≤d(i) then
14. *seed* ← *MERGE(seed*, $N_{i}(\text{seed}))$
15. merge ← 1
16. end if
17. end for
18. end while

In this algorithm, the top‐left most catchment basin, which owns the point at location (1, 1), is opted as the initial seed region. The catchment basins sharing watersheds with the seed are regarded as the seed's neighborhood regions. The resultant image of watershed transform can not effectively be used for computing a merge criterion (here, if r(i) ≤ d(i)), as most of the information is essentially lost in the oversegmented image. Therefore, catchment basins' information is referred from this image, but the merge criterion is examined using the data that is accessible from the original ROI. As a first step, a weighted graph G=(V,E) is constructed from the ROI, such that the ROI pixels are the vertices *V* and the edges *E* are defined between neighborhood pixels. The weight of an edge $w(v_{i},v_{j})$ is a measure of dissimilarity between $v_{i}$ and $V_{j}$. Assume there are n neighbors for the seed, say $N_{i}(\text{seed}),i∊[1..n]$. The intra‐region edge average (IRA) of the seed can be calculated as: The IRA of each of the seed's neighborhood is estimated in a similar fashion as it is done for the seed. The inter‐region edge mean (IRM) between the seed and any one of its neighbors *Nj(seed)* is calculated as:

(11) $$IRA(seed)=\frac{\sum_{\begin{array}{l} v_{i},v_{j}\in seed\\ (v_{i},v_{j})\in E \end{array}}w(v_{i},v_{j})}{\left|v_{a}\right|}$$

where

(12) $$v_{a}=\{(v_{i},v_{j})\in E|v_{i},v_{j}\in seed\}$$

The IRA of each of the seed's neighborhood is estimated in a similar fashion as it is done for the seed. The inter‐region edge mean (IRM) between the seed and any one of its neighbors $N_{i}(\text{seed})$ is calculated as:

(13) $$IRM(seed,N_{i}(seed))=\frac{\sum_{\begin{array}{l} v_{i}\in seed\\ v_{j}\in N_{i}(seed)\\ (v_{i},v_{j})\in E \end{array}}w(v_{i},v_{j})}{\left|v_{b}\right|}$$

where

(14) $$v_{b}=\{(v_{i},v_{j})\in E|v_{i}\in seed,v_{j}\in N_{i}(seed)\}$$

The dynamic threshold (DT) is computed as:

(15) $$DT(seed,N_{i}(seed))=\text{max}(IRA(seed)+\delta_{1},IRA(N_{i}(seed))+\delta_{2})$$

where

(16) $$\delta_{1}=\frac{C\times N_{R}}{\left|seed\right|}$$

(17) $$\delta_{2}=\frac{C\times N_{R}}{\left|N_{i}(seed)\right|}$$

Here, $N_{R}$ denotes the total number of catchment basins; *C* is a positive constant and is set to 1 for the experimental study; |seed| is the number of points in the seed; $\left|N_{i}(\text{seed})\right|$ is the number of points in the seed's neighborhood. For each neighborhood of the seed, the DT and IRM are estimated and, using this, the merge criterion is examined. If the merge criterion is met, then the two catchment basins (seed and its corresponding neighborhood) are united in the oversegmented image. In practice, merging (MERGE function as in the stated algorithm) can be attained by assigning the label of the seed to the label of its neighborhood catchment basin. If at least one merge occurs in the iteration, then the process is repeated with the updated seed. In the final result, the seed stands for the pectoral muscle and its complement over the final image constitutes the background (regions other than the pectoral muscle in the ROI).

## III. RESULTS & DISCUSSION

For testing, the weight of an edge was assigned the value of the absolute difference of the vertices that are connected by the edge. The standard validation criterion, the area normalized error,^(^ ^4^ ^,^ ^17^ ^,^ ^18^ ^)^ was used to analyze and compare the results obtained by this approach. This criterion involves the calculation of two error terms: false positive (FP) and false negative (FN). A pixel is assigned to FP when it is present in the algorithm‐identified pectoral muscle but not in the ground truth, and the pixel is considered as FN when it is present in the ground truth but not in the algorithm‐identified pectoral muscle. Mathematical representations of these two error terms, normalized by the area of the pectoral muscle, for a left MLO mammogram, are given by:

(18) $$FP=\frac{1}{A}\sum_{i=1}^{p}\text{max}[0,B_{\text{alg}}(i)-B_{gro}(i)]$$

(19) $$FN=\frac{1}{A}\sum_{i=1}^{p}\max[0,B_{gro}(i)-B_{\text{alg}}(i)]$$

where *A* is the area of the pectoral muscle in the ground truth and *p* is the number of rows of the pectoral muscle in the mammogram; $B_{\text{alg}}$ *(i)* is the horizontal coordinate of the pectoral muscle boundary point in the ith row as identified by the algorithm; $B_{\text{gro}}$ *(i)* is the horizontal coordinate of the pectoral muscle boundary point in the ith row of the ground truth. If there is an exact match between the ground truth and the algorithm identified pectoral muscle, then the value of FP and FN is zero. The mean of the error terms over “n” images can be calculated as:

(20) $$FP_{m}=\frac{1}{n}\sum_{i=1}^{n}FP_{i}$$

(21) $$FN_{m}=\frac{1}{n}\sum_{i=1}^{n}FN_{i}$$

In addition to FP and FN, the performance of the proposed method was also analyzed using the Hausdorff distance.^(28)^ The Hausdorff distance between a set of pixels in the ground truth (A) and a set of pixels of the algorithm identified pectoral muscle boundary (B) is defined as:

(22) H(A,B)=max(h(A,B),h(B,A))

where

(23) $$h(A,B)=\underset{p\in A}{\text{max}}\;\;\underset{q\in B}{\text{min}}\;\;D(p,q)$$

*D(p,q)* represents the Euclidean distance between the two points, *p* and *q*.

**Figure 5:**
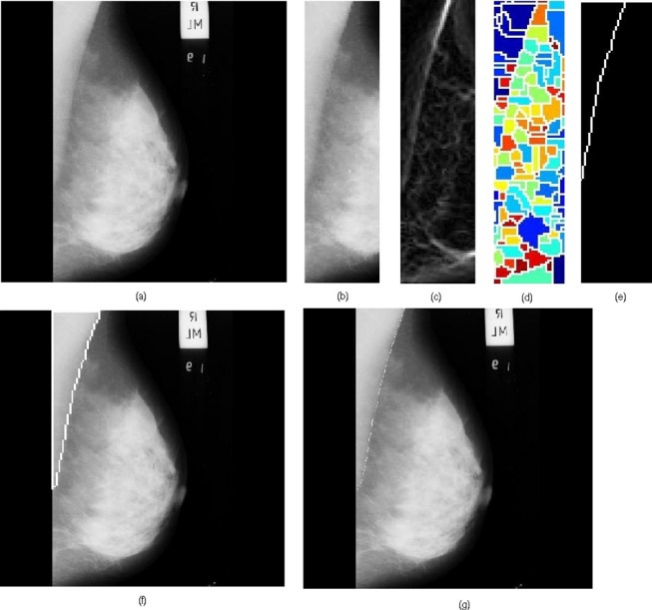
Results obtained for the image mdb004: (a) original mammogram, (b) ROI image, (c) ROI gradient, (d) watershed transformation of the ROI gradient, (e) identified pectoral muscle by the proposed merging algorithm, (f) identified pectoral muscle embedded over the original mammogram, (g) ground truth (pectoral muscle is marked white).

**Figure 6:**
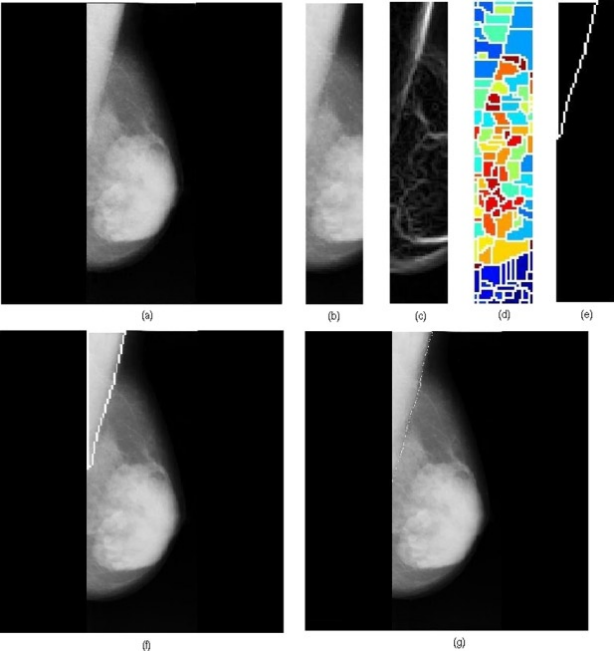
Results obtained for the image mdb037 where dense tissues appear near the pectoral muscle: (a) original mammogram, (b) ROI image, (c) ROI gradient, (d) watershed transformation of the ROI gradient, (e) identified pectoral muscle by the proposed merging algorithm, (f) identified pectoral muscle embedded over the original mammogram, (g) ground truth.

**Figure 7:**
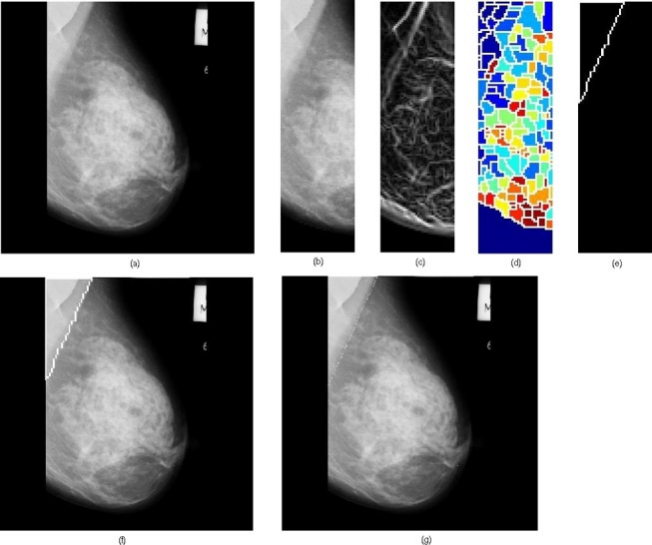
Results obtained for the image mdb125 where the multilayered pectoral muscle appears: (a) original mammogram, (b) ROI image, (c) ROI gradient, (d) watershed transformation of the ROI gradient, (e) identified pectoral muscle by the proposed merging algorithm, (f) identified pectoral muscle embedded over the original mammogram, (g) ground truth.

We processed a total of 84 mammograms and the performance of the proposed approach was evaluated. The proposed method took approximately 5 seconds to run in a computer featuring a Pentium IV, 3 GHz processor and 512 MB random access memory, with MATLAB 7.0 (The MathWorks, Natick, MA) computing environment. A few results are provided in Figs. 5, 6 and 7.

**Figure 8:**
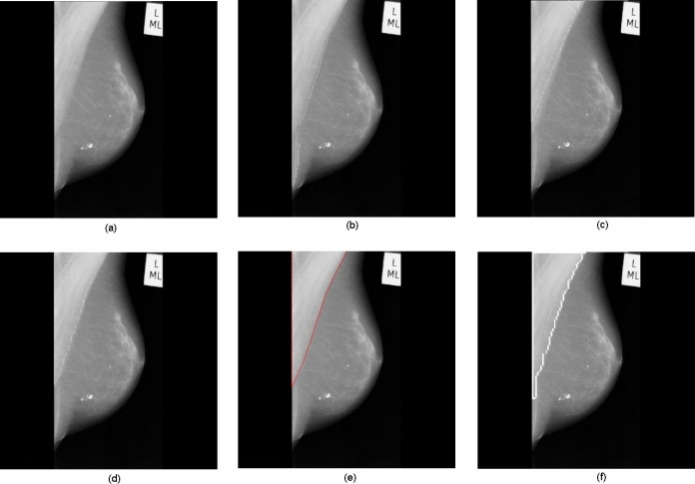
The longer pectoral muscle in the image mdb075 was underestimated by the proposed method: (a) original mammogram, (b) ground truth, and pectoral muscle boundary identified by the Hough transform (c), Gabor wavelets (d), the graph cut based merging method (e), and the proposed method (f).

**Figure 9:**
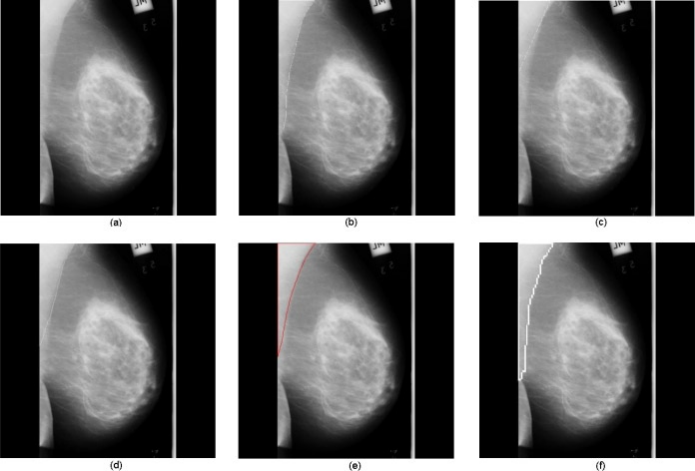
The longer pectoral muscle in the image mdb112 was correctly identified by the proposed method: (a) original mammogram, (b) ground truth, and pectoral muscle boundary identified by the Hough transform (c), Gabor wavelets (d), the graph cut based merging method (e), and the proposed method (f).

Figure 8 illustrates an example of underestimation of the longer pectoral muscle by the proposed method. The reason for underestimation is due to the drastic intensity discontinuity within the pectoral muscle in the image. However, in other mammograms, where the longer pectoral muscle regions are seen, the proposed method produced satisfactory results. An example of this can be seen in Fig. 9. It is important to note that other methods such as the Hough transform, Gabor wavelets, and the graph cut based merging method underestimated the pectoral muscle in both these mammograms (mdb075 and mdb112), which is evident from the Figs. 8 and 9.

The summary of the results obtained for the proposed approach, and for other state‐of‐the‐art methods, is given in Table 1. The widely accepted error measurement criterion (FP and FN) in the field of medicine was used to compare all the reported methods. For the Hough transform,^(11)^ the mean false positive is considerably closer to the lowest value. But the mean false negative is higher than all other methods. Also, the anticipated accuracy, in terms of the number of results with smaller errors, is not attained; approximately 79% of results lie in the greatest error range. For Gabor wavelets,^(4)^ the mean false negative is better than all of the compared methods. The mean false negative is also considerably good. It gained higher‐quality results holding smaller errors in about 54% of the mammograms. At the same time, in many

**Table 1:** Segmentation performance analysis by area normalized error. The first two rows represent the mean false positive and mean false negative values. The remaining rows report the distribution of the false positive and false negative values. For all the methods except the MST algorithm, the values reported are based on 84 mammograms. For the MST algorithm, the values reported are based on 82 mammograms (as the method failed to identify the pectoral muscle in two mammograms).

	*Hough Transform*	*Gabor Wavelets*	*Adaptive Pyramid*	*Minimum Spanning Tree*	*Graph Cut Based Merging*	*Proposed Approach*
${\text{FP}}_{m}$	0.0198	0.0058	0.0371	0.0255	0.0064	0.0085
${\text{FN}}_{m}$	0.2519	0.0577	0.0595	0.1168	0.0558	0.0488
FP<0.05 & FN<0.05	10	45	50	40	43	46
min(FP,FN)<0.05 & 0.05<max(FP,FN)<0.10	0	0	18	20	19	25
min(FP,FN)<0.05 & max(FP,FN)>0.10	0	0	11	18	22	13
0.05<FP<0.10 & 0.05<FN<0.10	8	22	0	0	0	0
0.05<min(FP,FN)<0.10 & max(FP,FN)>0.10	0	0	0	1	0	0
FP>0.10 & FN>0.10	66	17	5	3	0	0

other mammograms, the expected accuracy is not reached. For the AP algorithm,^(17)^ the mean false negative is comparable to the mean false negative of many other methods. The method resulted in smaller errors in a higher percent of the mammograms, while the method produced poor quality results in approximately 6% of the mammograms. A good distribution of the error terms can be noticed for the MST algorithm;^(17)^ apart from this, the results are not as good as many other methods. The mean false positive, mean false negative, and the distribution of error terms are in an acceptable range for the graph cut based merging method.^(18)^

The mean false negative of the proposed approach is better than all of the compared methods. At the same time, its mean false positive is closer to the lowest one. The error terms' distribution is also satisfactory. The segmentation results are of higher quality in about 55% of the mammograms as both the error terms are less than 0.05. In about 30% of the mammograms, any one of the error terms is less than 0.05, while the other term has the error value between 0.05 and 0.10. One of the error terms is less than 0.05, while the other error term's value is greater than 0.10 in approximately 15% of the mammograms. Moreover, the fact that zero values populated for the number of images at higher error ranges clearly shows the acceptable precision of the proposed approach.

The mean and standard deviation of the Hausdorff distance for the proposed method over 84 mammograms were determined as 3.85±1.07 mm. For the same dataset, the mean and standard deviation for the Hough transform and Gabor wavelets were previously reported by Ferrari et al.^(4)^ as 7.08±5.26 mm and 3.84±1.73 mm, respectively. The mean of the proposed method and the mean of Gabor wavelets are, respectively, approximately equal and considerably less than the mean of the Hough transform. However, with respect to the standard deviation, it is evident that the consistency of the proposed method is better than the Hough transform and Gabor wavelets.

## IV. CONCLUSIONS

The method described here for pectoral muscle identification in MLO mammograms uses watershed transformation and the proposed merging algorithm. When mammograms were treated with the watershed transformation, they exhibited some important properties that were used to identify the pectoral muscle in mammograms. One of these properties is the existence of a unique watershed line corresponding to the pectoral muscle boundary. If a few points lying on this watershed line are known, either automatically or semi‐automatically, then the complete pectoral muscle could be traced by proper traversing of the line. The proposed merging algorithm was used to obtain the actual pectoral muscle boundary by uniting irrelevant catchment basins of the oversegmented pectoral muscle region. This new merging algorithm chooses a pectoral muscle region as the initial seed by using one of the attributes of the pectoral muscle. The neighbors of the seed are merged with the seed, if their homogeneity is comparable to the seed. Finally, the pectoral muscle boundary is inferred from the seed. The validation results clearly demonstrate the improved performance of the proposed approach in terms of accuracy. The mean false positive of the proposed approach is comparable to that of the state‐of‐the‐art methods. At the same time, the obtained mean false negative is better than that for the existing methods. The consistency of the proposed method is also satisfactory, which is apparent from the measured low Hausdorff distance. These results clearly demonstrate that this approach can be effectively used as a preprocessing step in the detection of breast cancers in CAD.

## ACKNOWLEDGMENTS

The authors would like to thank R.M. Rangayyan for providing a set of mammograms with radiologist‐drawn pectoral muscle boundaries.
